# Has Deep Brain Stimulation Shown Its Full Potential in Treatment-Resistant Depression? A Scoping Review

**DOI:** 10.1016/j.bpsgos.2025.100682

**Published:** 2025-12-23

**Authors:** Liene Puke, Joelle Rosselet Amoussou, Armin von Gunten, Julien Elowe

**Affiliations:** a Division of Adult Psychiatry and Psychotherapy West, Department of Psychiatry, Lausanne University Hospital and University of Lausanne, Lausanne, Switzerland; b Medical Library-Cery, Lausanne University Hospital and University of Lausanne, Lausanne, Switzerland; c Service of Geriatric Psychiatry and Interventional Psychiatry, Department of Psychiatry, Lausanne University Hospital and University of Lausanne, Lausanne, Switzerland

**Keywords:** Deep brain stimulation, Deep brain stimulation targets, Major depressive disorder, Personalized treatment in psychiatry, Treatment-resistant depression

## Abstract

Major depressive disorder is increasingly conceptualized as a networkopathy involving dysfunction across brain networks rather than isolated regions. This perspective has supported the use of deep brain stimulation (DBS) in treatment-resistant depression (TRD), where conventional therapies have failed. In this scoping review, we examined peer-reviewed studies on bilateral DBS for TRD, with a focus on the relationship between stimulation targets, conceptual frameworks of depression, and clinical outcome measures. A comprehensive literature search was conducted in September 2024 across 6 bibliographic databases, supplemented by citation tracking strategies to identify additional relevant studies. Following Preferred Reporting Items for Systematic Reviews and Meta-Analyses (PRISMA) 2020 guidelines, we screened and selected studies based on predefined eligibility criteria. The review identified significant variability in how TRD is defined, which brain targets are selected, and how these are associated with specific symptom dimensions. Anatomical targets varied widely, reflecting differing neurobiological rationales. While most studies assessed symptom severity using standardized scales such as the Montgomery–Åsberg Depression Rating Scale or Hamilton Depression Rating Scale, a minority of studies (8 of 48 [16.7%]) did not specify which symptom dimensions were expected to respond to DBS. Despite methodological heterogeneity, DBS appears promising for symptom alleviation in TRD. However, its clinical benefits remain to be fully established. The review highlights the need for greater standardization, including consistent definitions of TRD, clear symptom mapping, and improved integration of patient-reported and functional outcomes. Although most existing studies focus on bilateral stimulation, future work should also explore unilateral and multitarget approaches to advance toward more personalized neuromodulation strategies.

## Plain Language Summary

In this scoping review, we examined the application of deep brain stimulation (DBS) in treatment-resistant depression (TRD), focusing on the relationship between stimulation targets, conceptual frameworks of depression, and clinical outcome measures. The review identified substantial variation in how TRD is defined, which brain targets are selected, and how these are associated with specific symptom dimensions. While the potential of DBS has been demonstrated, its effectiveness remains inconclusive. The findings highlight the need for standardized definitions, improved symptom-target mapping, and greater integration of patient-reported and functional outcome measures to strengthen the assessment and personalization of DBS interventions in TRD.

Major depressive disorder (MDD) is increasingly being conceptualized as more than a mere disorder of a single brain region, involving dysfunctional brain circuits that themselves interact within large-scale networks (1,2). More than 25 years ago, Mayberg proposed a model suggesting that suppressing ventral paralimbic regions normalizes underactive dorsal neocortical areas, with rostral cingulate integrity being essential for this response (3). This model broadened the understanding of MDD, shifting from localized abnormalities to an integrative view of widespread structural and functional brain dysfunctions. Subsequent studies have supported the perspective of a networkopathy to better define MDD’s heterogeneous symptoms and guide new targeted therapies (4, 5, 6, 7, 8, 9, 10).

Psychiatry is actively embracing personalized approaches, especially in MDD, where precision psychiatry leverages genetic, biological, and psychosocial markers to guide individualized treatment strategies (11, 12, 13, 14). While many patients respond to antidepressants and psychotherapy, approximately 30% experience treatment-resistant depression (TRD) (15, 16, 17). Patients with TRD face poorer clinical outcomes, reduced quality of life, and lower work productivity compared with other patients with MDD (18,19). Although some new pharmacological options for TRD have emerged (20), deep brain stimulation (DBS) has been attracting growing interest since the early 2000s as a promising nonpharmacological alternative for refractory cases (21,22). Mayberg’s model has influenced the selection of brain targets by focusing on circuits associated with the 2 core symptoms of MDD: negative affect and anhedonia. For negative affect, key targets include the subcallosal cingulate cortex (SCC) and lateral habenula (LHb), while targets such as the ventral capsule/ventral striatum (VC/VS), medial forebrain bundle (MFB), and inferior thalamic peduncle (ITP) are utilized for anhedonia (23).

DBS has shown promise for patients unresponsive to conventional treatments, but it remains unapproved for TRD due to mixed outcomes (17,24). First, open-label studies have reported that up to 90% of patients who failed psychotherapy or medication benefited from DBS (24, 25, 26), but randomized controlled trials have yielded less consistent results (24,27, 28, 29). Rabin *et al.* (17) suggested that inadequate outcome measures may explain this variability. Common scales, such as the Montgomery–Åsberg Depression Rating Scale (MADRS) (30) and Hamilton Depression Rating Scale (HDRS) (31), provide only partial representations of TRD symptoms and overlook patient-prioritized outcomes, such as reduced negative thinking and improved self-confidence (17,32, 33, 34). Outcome measures should also evolve beyond symptom reduction to include quality of life, social participation, and interpersonal functioning, aligning with patient goals (35). Second, there is no universal definition of TRD. While a recent Delphi-method consensus defines TRD as <25% improvement with at least 2 adequate antidepressant trials (36), this is not consistently applied across studies. Finally, research on DBS parameters, including stimulation frequency and pulse width, has been limited but suggests that these settings are critical for optimizing efficacy (23). Therefore, addressing these methodological and definitional gaps could be essential to establish DBS as a reliable TRD treatment, especially as DBS conceptualization through a target symptom framework could offer a promising path toward precision neuromodulation.

Accordingly, current limitations in the appropriate evaluation of DBS performance in TRD may underestimate its real effectiveness (17,37, 38, 39). Therefore, in this scoping review, we intend to map the research done in the field of bilateral DBS in TRD based on the different definitions of TRD, the various commonly used DBS targets, and the depression models from which they are derived.

## Objectives

Our objective was to systematically identify and summarize peer-reviewed, published literature reporting bilateral DBS for TRD and aimed at understanding the current state of knowledge in the field and key characteristics of TRD. We did not set any limits in terms of the time window of the studies. The review addresses 3 research issues: First, in order to assess the homogeneity of the chosen definition of TRD, we examined the clinical and psychometric characteristics retained in the definition of TRD in DBS studies. Second, given the symptomatic heterogeneity of TRD, we attempted to identify whether DBS was conceptualized from a target symptom framework perspective. Third, we aimed to identify DBS parameters and corresponding clinical outcome measures.

## Methods

### Protocol and Registration

We registered our study protocol on December 29, 2022 (https://osf.io/8hmy5/), on the Open Science Framework, a web application providing connection and support for the research workflow to collaborate; document; archive; share; and register research projects, materials, and data. This scoping review was carried out in accordance with the Joanna Briggs Institute (JBI) manual for scoping reviews (40). The Preferred Reporting Items for Systematic Reviews and Meta-Analyses extension for Scoping Reviews (PRISMA-ScR) standards were followed for reporting (41,42).

### Eligibility Criteria

We considered all types of research design and articles with no language limitation and no time window limitation. We applied the Population (or participants)/Concept/Context (PCC) framework recommended by JBI to identify the main concepts in our primary review questions. To be included in this scoping review, articles had to focus on patients with TRD and the application of multiple (bilateral) DBS targets. Peer-reviewed journal articles were included if they matched the PCC framework. Articles were excluded if they did not match the conceptual framework by using the following exclusion criteria: animal studies; electrophysiological studies; case reports; editorials, letters, personal views, book reviews, reports, theses, conference proceedings, white papers, clinical guidelines, and internal documents (gray literature); and studies focusing exclusively on other pathologies than TRD (such as obsessive-compulsive disorder).

### Search Strategy and Information Sources

A comprehensive literature search was conducted in September 2024 in collaboration with a medical librarian (JRA) in 6 bibliographic databases: MEDLINE ALL (Ovid), Embase, APA PsycInfo (Ovid), Web of Science Core Collection, the Cochrane Database of Systematic Reviews, and the Cochrane Central Register of Controlled Trials. The searches were performed without language or date restrictions. Deduplication of references exported from the databases was performed by Deduklick (Risklick AG) (43). Backward and forward citations searches were performed through the use of Citationchaser (44) and Web of Science Core Collection, based on the studies that were included, did not reveal any new records of interest.

Details regarding the search syntax, keywords, and index terms used are provided in Table 1.

**Table 1:** TRD Characteristics

No.	Study	Response to Pharmacotherapy, Augmenting/Combination Agents	Lifetime MDD Episodes	Length of Current Depressive Episode	ECT, No. of Sessions	Evidence-Based Psychotherapy	Psychiatric Comorbidity	Current Psychotropic Medication
1	Schlaepfer *et al.*, 2008 (94)	Patient 01: 7 ADs failed, female, 5 NL	6	17 mo	13 bilateral ECT treatments	Yes, but no response	No	90 mg duloxetine, 1 mg risperidone, 10 mg diazepam, 75 mg L-thyroxine, 75 mg melperone
		Patient 02: 17 ADs failed, male, 5 NL, Li	2	9 y	4 courses of ECT with 10 treatment sessions	Yes, but no response	No	50 mg quetiapine, 75 mg amitriptyline
		Patient 03: 8 ADs failed, male, 3 NL, Li	2	9 y	2 treatment courses, 10 unilateral and 15 bilateral treatment sessions	Yes, but no response	No	50 mg quetiapine, 150 mg amitriptyline, 75 mg L-thyroxine
2	Bewernick *et al.*, 2010 (50)	No. of past medical treatment courses: mean 20.8 (SD = 8.4) Failure to respond to adequate trials (>5 wk at the maximum recommended dose, ≥3 different classes of AD ATHF, >3 adequate trials of augmentation/combination of a primary AD using at least 2 different agents): mean 8.3 (SD = 3.2)	At least 4 episodes of MDD or chronic over 2 y	At least 5 y after first episode of MDD Mean length of the current episode: 10.8 (SD = 7.5) y	Lifetime ECT treatments mean 20.8 (SD = 8.6)	No response Mean 316.4 h (SD = 265.2) >20 sessions	30%	Stable drug regimen at least 6 wk before study
3	Bewernick *et al.*, 2012 (51)	On average, 7.9 medical treatment courses with an ATHF score >3 defining an adequate treatment dose and length, including augmentation and combination therapy No. of past medical treatment courses: 22.18 (SD = 7.73) No. of medications included in formula: 13.82 (SD = 5.34)	No. of previous episodes (lifetime): 1.29	Mean length of the current episode: 9.26 (SD = 7.64) y	Past ECT/MST 21.09 (SD = 8.48) >6 bilateral treatments	Mean 263.64 (SD = 263.88) >20 sessions	No	At the time of implantation, the mean no. of AD medications was 4.3. Drug free or on stable drug regimen at least 6 wk before study entry
4	Grubert *et al.*, 2011 (52)	No. of ADs at implant: mean 4.3 (SD = 1.34) No. of past medical treatment courses: 20.8 (SD = 8.35) No. of medications included in formula: 14.1 (SD = 5.63) Mean total of ATHF score: 41.7 (SD = 15.33)	No. of previous episodes (lifetime): 1.6 (SD = 0.89)	Length of current episode: 10.79 (SD = 7.51) y	20.8 (SD = 8.63)	Mean 316.4 h (SD = 265.25)	30%	Pharmacotherapy and psychotherapy were kept constant throughout 6 mo
5	Millet *et al.*, 2014 (53)	Thase and Rush stage V	≥3	>2 y, mean 3.3 (SD = 2.7)	Yes	Yes	50%	NS
6	Schlaepfer *et al.*, 2013 (55)	No. of AD at implant: mean 4.0 (SD = 3.9) Total ATHF score current episode: mean 65.0 (SD = 12.4) No. of treatment trials with ATHF >3, medical treatment courses: mean 14.0 (SD = 3.5) Failure to respond to adequate trials of primary AD from ≥3 different classes, adequate trials of augmentation/combination of a primary AD using ≥2 different agents	≥4 episodes of MDD or chronic over 2 y	>5 y after first episode of MDD Mean 7.6 (SD = 5.0) y	Mean 14.3 (SD = 14.3) unilateral	Yes, but no response >20 sessions	No	Drug free or on stable drug regimen at least 6 wk before study
7	Coenen *et al.*, 2019 (54)	Treated on average with 18.9 ADs (SD = 10.3) ATHF score for the current episode was ≥3.	NS	Current depressive episode 10.3 y duration on average (SD = 9.2)	Received 20 ECTs without response on average	On average, 70 h without response	No	Medication was left constant at least 8 wk before and after surgery
8	Fenoy *et al.*, 2018 (56)	Refractory to >6 wk of multiple medication regimens	Recurrent >4 episodes	>2 y 5 y since the onset of the fist depressive episode	≥6 bilateral ECT treatments	>20 sessions psychotherapy	No	NS
9	Fenoy *et al.*, 2016 (57)	Refractory to >6 wk of multiple medication regimens	Recurrent >4 episodes	>2 y 5 y since the onset of the first depressive episode	≥6 bilateral ECT treatments	>20 sessions psychotherapy	No	NS
10	Bewernick *et al.*, 2018 (58)	Failure to respond to adequate trials of primary ADs from ≥3 different classes, adequate trials of augmentation/combination of a primary AD using ≥2 different agents	≥4 episodes of MDD or chronic episodes over 2 y, >5 y after the first episode of MDD	8.57 (SD = 7.75) y	Yes >6 bilateral ECT sessions	>20 sessions CBT, CB analysis system of psychotherapy	No	Drug free or on stable drug regimen at least 6 wk before study entry
11	Kilian *et al.*, 2024 (59)	3 different classes of AD augmentation/combination therapy of primary AD with other agents	At least 4	2 y	ECT >6 sessions	Individual psychotherapy >20 h	No	NS
12	Merkl *et al.*, 2013 (60)	Current pharmacotherapy: Yes ATHF 13–20	4–20	NS	No. of ECTs vary from 0 to >100 ECT lifetime	59 h on average	No	Concomitant medication stable for 6 wk before and after surgery; SNRI 100%, SSRI 75%, nonselective α_2_-adrenoreceptor antagonist 30%, atypical NL 50%, Li/pregabalin/mood stabilizers 30%, nonhydrazine reversible MAOI 16%, ketamine injections 30%, BZ 50%
13	Kennedy *et al.*, 2011 (79)	DSM-IV-TR MDD No response to ≥4 adequate treatment trials, no. of medications: mean 4.2 (SD = 4.1)	Lifetime no. of MDD: mean 3.9 (SD = 3.1)	>1 year Mean 6.9 (SD = 5.6) y	Yes	Yes	NS	NS
14	Conroy *et al.*, 2021 (68)	MDD or BD in current treatment-resistant MDE. Failure to respond to ≥4 different AD medications, evidence-based ECT and psychotherapy 2 patients with BD II, 3 patients with MDD	NS	Mean 43.4 (SD = 16.8) mo	Mean 5.6 (SD = 2.3)	Yes	No	Stable dose of psychiatric medication, consistent throughout the study
15	Lozano *et al.*, 2008 (71)	Failure to respond to ≥4 different treatments, including AD pharmacotherapy of sufficient dose and duration, evidence-based psychotherapy, and ECT	Mean 3.9 (SD = 3.1)	At least 1 y	17/20 received	20/20 received	No	NS
16	Mayberg *et al.*, 2005 (88)	Failure to respond to ≥4 different AD treatments, including medications, evidence-based psychotherapy, or ECT	Mean 4.7 (SD = 5.0)	Mean 5.6 (SD = 3.0) y	5/6 patients had ECT treatment.	6/6 received	No	Patient 1: SSRI/SNRI, bupropion, atypical antipsychotic, BZ, stimulant; patient 2: SSRI/SNRI, atypical antipsychotic; patient 3: SSRI/SNRI, bupropion, atypical antipsychotic, BZ; patient 4: SSRI/SNRI, bupropion, BZ, mood stabilizer, other; patient 5: SSRI/SNRI, other; patient 6: SSRI/SNRI, BZ, stimulant, other
17	Holtzheimer *et al.*, 2017 (73)	Failure to respond to 4 adequate AD treatments, including ≥3 medications from 3 different classes	NS	≥12 mo	Yes, NS	Yes, but no response	No	NS
18	Holtzheimer *et al.*, 2012 (102)	Total no. of treatments lifetime: mean 24.1 (SD = 10.6)	No. of depressive episodes lifetime: mean 6.9 (SD = 9.3)	Mean 64.1 (SD = 53.7) mo	Prior ECT, failed or intolerant: 94%	Yes, 100%, but no response	Two patients eating disorder, 1 panic disorder, 1 generalized anxiety disorder	Patients were taking a mean of 3 (SD = 2) psychotropic medications at the time of surgery.
19	McInerney *et al.*, 2017 (53)	Failure to respond to ≥4 different treatments No. of medications: mean 4.2 (SD = 4.1)	Mean 3.9 (SD = 3.1)	Duration of MDE mean 6.9 (SD = 5.6) y	ECT, yes 85% (*n* = 17)	Yes, 100%	No	No. of medications: mean 4.2 (SD = 4.1) 2 patients BZ, 2 patients 1 AD, 5 AD + AP agent or BZ, 11 patients 2 ADs from 2 different classes + Li or atypical AP and a BZ
20	Eitan *et al.*, 2018 (74)	Resistance to a ≥4 adequate depression treatments from ≥3 different treatment categories ATHF ≥3	First episode onset before age 45	>12 mo	Yes	Yes	NS	All patients maintained AD medication and psychotherapy, did not receive new medication, did not increase current AD dose
21	Merkl *et al.*, 2018 (60)	ATHF ≥3	3–10, average 5	>2 y	0–100 No. of ECT lifetime, average 29	Yes	1 patient BPAD I	Patient 1: MAOI, Li, AP Patient 2: AP, zopiclone Patient 3: BD Patient 4: SNRI Patient 5: NaSSa Patient 6: Pregabaline, AP, agomelatine, Patient 7: BD, SSRI Patient 8: BD
22	Riva-Posse *et al.*, 2018 (61)	Current depressive episode ≥12 mo without significant response to ≥4 adequate AD treatments ATHF ≥3	3.82 (SD = 1.47)	>12 mo	Lifetime failure or intolerance	All received CBT	No	Medication changes were not allowed during the preoperative evaluation phase or the initial 24 wk of chronic DBS Medication in current episode: mean 7.18 (SD = 1.83)
23	Ramasubbu *et al.*, 2015 (62)	Failure to respond to 4 different classes of AD, psychotherapy, and ECT despite adequate dosage, duration, and compliance with treatment	NS	NS	Yes	Yes	NS	Patient 1: citalopram 60 mg/day, clonazepam 0.5 mg, lithium carbonate 600 mg, quetiapine 150 mg. Patient 2: duloxetine 60 mg/day, zopiclone 22.5 mg, gabapentin 1200 mg/day, clonazepam 1.5 mg/day, Synthroid 0.15 mg/day Patient 3: no ADs, Dexedrine Xr 10 mg/day, risperidone 0.75 mg, mirtazapine 11.25 mg, aripiprazole 6 mg
24	Bogod *et al.*, 2014 (63)	Fail to respond to ≥4 different classes of AD and psychotherapy	Mean 3 episodes	Mean 8.3 y	75% yes	100% yes	No	Yes
25	Accolla *et al.*, 2016 (75)	Failure to respond to complex pharmacotherapy and ECT, ATHF: mean 18	Mean 5 episodes	NS	ECT lifetime mean = 21	Yes	No	Concomitant medication was kept stable for 6 wk prior to and after surgery Patient 1: pregabaline, agomelatine, quetiapine, levothyroxine Patient 2: Li, trancypromine, pregablaine, quetiapine Patient 3: no medication Patient 4: zopiclone, quetiapine, trimipramine Patient 5: mirtazapine
26	Clark *et al.*, 2020 (104)	16 with chronic TRD	NS	Mean 25.5 (SD = 10.94) mo and 28.6 (SD = 6.75) mo	NS	NS	NS	NS
27	Ramasubbu *et al.*, 2013 (65)	Failed 4 different classes of AD including augmentation or combination strategies with Li, atypical AP, anticonvulsants, and AD	NS	NS	Yes	Yes	No	NS
28	Crowell *et al.*, 2019 (66)	No response to ≥3 AD treatments (≥4 lifetime treatment failures)	Mean 5.7 (SD = 7.3)	Mean 53.2 (SD = 45.5) mo	96% (*n* = 27)	Yes, all	NS	NS
29	Elias *et al.*, 2022 (67)	NS	NS	Disease duration at surgery: mean 22.2 (SD = 9.5) mo	NS	NS	Yes	NS
30	McCall *et al.*, 2020 (76)	Failed to respond to ≥4 AD treatments ATHF ≥3	3.82 (SD = 1.47)	≥12 mo	Lifetime failure or intolerance	All received CBT	No	Yes
31	Aibar-Durán *et al.*, 2022 (second part of the study) (103)	Refractory to multiple adequate second-generation AD trials	NS	Mean 26.5 (range 8–34) y	Yes	Yes	No	Yes
32	Clark *et al.*, 2020 (104)	No response to treatment more than 1 y No. of past medications: mean 22.8 (SD = 2.6) Ketamine 18% TRD stage IV or V 82% TRD severity mean: 12.7 (SD = 0.4)	NS	24 (SD = 0.4) mo	86%	CBT 12 wk	No	No. of current medications: 3.4 (SD = 0.4)
33	Conen *et al.*, 2018 (105)	Mean no. of different pharmacological treatments 18.5 ADs from 3 different classes Li augmentation	Mean 3.5 lifetime episodes	11.5 (SD = 10) y	≥2 courses of ECT 43 mean received treatments	CBT	Yes	Mean 4.5 current psychotropic treatments
34	Williams *et al.*, 2016 (47)	Had not benefited from trials of ≥4 classes of AD medication, ATHF mean 5.8 (SD = 2.05) No. of psychiatric treatments in current depressive episode: mean 9.8 (SD = 5.3)	NS	Mean 46 (SD = 53.7) mo	Yes	Yes, ≥6 wk	2 patients with BPAD	No. of psychotropics at baseline: mean 6 (SD = 2.23) No. of psychotropics at 5 y: 4.4 (SD = 1.34)
35	Nahas *et al.*, 2009 (46)	MDE as part of either BD or MDD, scored >20 on the 24-item HDRS, failure to respond to an adequate trial of 4 classes of AD	NS	42.8 (SD = 38.3) mo	ECT, 3/5	Yes, all, ≥6 wk	NS	NS
36	Zhang *et al.*, 2022 (21)	MDD or BD, chronic illness episode for >2 y or a recurrent illness with ≥4 lifetime episodes. HDRS-17 score ≥18 Failure to respond to multiple classes of medication, ≥2 types of medications for MDD, and ≥3 types for BD	4.0 (SD = 2.0)	7.0 (SD = 6.0) y	5/7	3/7	3/7	Yes
37	Fitzgerald *et al.*, 2018 (77)	No. of AD trials: 11–20 Duration of depression episodes: 10–18 mo. Also Li, thyroid hormone, and atypical AP, AD combination therapies	2–4	4 to >10 y	1–6	All CBT, ≥10 sessions over 3 mo	No	Yes, BZ and quetiapine as well as AD
38	Malone *et al.*, 2010 (106)	Failure to respond to ≥5 courses of medication ≥5-y history of chronic or recurrent depression	NS	≥2 y	≥1 trial of ECT, ≥6 bilateral treatments	Yes	No	NS
39	Malone *et al.*, 2009 (107)	≥5-y history of chronic or recurrent depression Adequate trials of >6 wk at maximum recommended or tolerated dose of primary AD drugs from ≥3 different classes, >4 wk of augmentation/combination strategies using primary AD with ≥2 other different agents	NS	≥2 y	≥1 trial of ECT, ≥6 bilateral treatments	≥20 sessions with an experienced therapist	No	Stable regimen of psychotropic medication for ≥6 wk before study entry
40	Dougherty, *et al.*, 2015 (108)	Lack of clinically substantive response to ≥4 adequate trials of AD therapy Three of 4 trials occurred in current episode. One of 4 trials had to have included combination of ≥2 ADs from 2 different classes. One of the augmentation agents: Li, triiodothyronine, buspirone, pindolol, or an atypical AP ATHF mean of 10.8 (SD = 3.2) Augmentation therapy failed mean 8.6 (SD = 3.2) treatments during current depressive episode	No. of episodes: mean 2.8 (SD = 1.8)	>2 y	Complete course of ECT Only 34% achieved any benefit.	>6 wk of psychotherapy for the current and previous episodes without significant improvement; 16 participants were currently receiving the therapy	No	Psychiatric treatment regimen kept for >30 days prior to the study screening MADRS for inclusion
41	Kubu *et al.*, 2017 (109)	Lack of clinically substantive response to ≥4 adequate trials of AD therapy Three of four trials occurred in current episode. One of 4 trials had to have included a combination of ≥2 ADs from 2 different classes. One of the augmentation agents: Li, triiodothyronine, buspirone, pindolol, or an atypical AP. ATHF mean of 10.8 (SD = 3.2) Augmentation therapy failed: mean 8.6 (SD = 3.2) treatments during current depressive episode	No. of episodes mean 2.8 (SD = 1.8)	More than 2 y	Complete course of ECT Only 34% achieved any benefit.	>6 wk of psychotherapy for the current and previous episodes without significant improvement; 16 participants were currently receiving the therapy.	No	Psychiatric treatment regimen kept for >30 days prior to the study screening MADRS for inclusion
42	Hitti *et al.*, 2021 (110)	Failed ≥4 adequate medication trials, one half failure to respond to VNS (removed before DBS)	NS	NS	Yes	NS	NS	NS
43	Bergfeld *et al.*, 2016 (111)	Failure to ≥2 different classes of second-generation AD, 1 trial of a tricyclic AD, 1 trial of a tricyclic AD + Li augmentation, 1 trial of an MAOI No. of past medications: mean 10.8 (SD = 3.3)	TRD episodes, No. (%) 1 (40%) 2 (12%) >2 (48%)	>2 y	≥6 sessions of bilateral ECT No. of past ECT sessions: mean 68.9 (SD = 103.6)	NS	No	NS
44	Raymaekers *et al.*, 2017 (95)	Failure to respond to the pharmacotherapy	Recurrent >4 episodes	>2 y 5 y since the onset of the first depressive episode	Failure to ECT	NS	In 6/7 patients (86%)	Medication tapered off to a bearable minimum and maintained on a stable regime throughout the first year of DBS
45	Van der Wal *et al.*, 2020 (49)	Patients were eligible if they failed to respond to ≥2 classes of second-generation ADs in adequate dosage, 1 trial of TCA with subsequent Li augmentation, 1 trial of MAOI	For 10 patients, 1 episode For 3 patients, 2 episodes For 12 patients, >2 episodes	Mean 83.8 (SD = 76.2) mo and mean 89.0 (SD = 81.1) mo	No. of past ECT series: mean 2.3 (SD = 1.7) and mean 2.2 (SD = 1.4) No. of past ECT sessions: mean 68.9 (SD = 103.6) and 70.7 (SD = 112.1)	NS	No	NS
46	Bergfeld *et al.*, 2022 (48)	Failure of ≥2 distinctly different classes of second-generation ADs (SSRI, SNRI), 1 trial of TCA, and 1 trial of TCA with Li and 1 trial with MAOI	For 11 patients, 1 episode For 3 patients, 2 episodes For 11 patients, >2 episodes	Mean 83.8 (SD = 76.2) mo	≥6 sessions of bilateral ECT	NS	No	NS
47	Ramasubbu *et al.*, 2020 (112)	No response to treatment more than 1 y No. of past medications: 22.8 mean (SD = 2.6) Ketamine 18% TRD stage IV or V 82% TRD severity mean 12.7 (SD = 0.4)	NS	24 (SD = 0.4) mo	86%	CBT 12 wk	No	No. of current medications: mean 3.4 (SD = 0.4)
48	Wang *et al.*, 2024 (45)	No response to ≥3 adequate trials of AD therapy, including AD and psychotherapy and ECT	≥4 episodes	≥24 mo	Yes	Yes	No	Maintained stable

AD, antidepressant; AP, antipsychotic treatment; ATHF, Antidepressant Treatment History Form; BD, bipolar disorder; BPAD, bipolar affective disorder; BZ, benzodiazepines; CBT, cognitive behavioral therapy; ECT, electroconvulsive therapy; HDRS-17, 17-item Hamilton Depression Rating Scale; Li, lithium; MAOI, monoamine oxidase inhibitors; MDD, major depressive disorder; MDE, major depressive episode; NaSSA, noradrenergic and specific serotonergic antidepressants; NL, neuroleptic treatment; NS, not specified; SNRI, serotonin and norepinephrine reuptake inhibitor; SSRI, selective serotonin reuptake inhibitor; T3, triiodothyronine; TCA, tricyclic antidepressants; TRD, treatment-resistant depression; VNS, vagus nerve stimulation.

### Selection of Sources of Evidence

The Rayyan platform was used to collect studies, with 2 reviewers (LP and JE) screening publications independently. Disagreements were resolved through discussion with other reviewers (JE and AvG). Data from eligible articles were carefully reviewed. Case studies and gray literature were excluded as the review focused on specific research questions in DBS for TRD.

### Data Charting Process and Synthesis of Results

Three reviewers (LP, JE, and AvG) developed a data charting form to extract relevant variables, with 2 reviewers (LP and JE) updating it through discussion to resolve disagreements. Data from eligible studies were manually charted into 2 tables. The first captured TRD characteristics, including treatment history (pharmacotherapy, electroconvulsive therapy [ECT], psychotherapy), number of MDD episodes, comorbidities, and current medications (Table 1). The second summarized general and study-specific information (country, conceptual frameworks of depression, clinical targets, DBS procedure, outcomes, and response rate) (Table 2). The label NS or not specified was applied to relevant characteristics (Tables 1 and 2) where the authors did not explicitly state, for example, “the conceptual framework of depression” or “clinical or symptomatic target” or when such specification was not the primary focus of the study.

**Table 2:** Characteristics of DBS Studies, Summarizing General and Study-Specific Information

No.	Study	Country	Conceptual Framework of Depression	No. of Patients	Clinical and/or Symptomatic Target(s)	DBS, Target	Procedure, Parameters, Modality, Duration	Depression Measures as Primary Outcome
1	Schlaepfer *et al.*, 2008 (94)	Germany	Dysfunction of reward and motivation processing systems, anatomical connectivity to limbic and prefrontal regions, incl. Cg25	3	Anhedonia, brain metabolism	NAc	Modality: bilateral Frequency: 145 Hz Amplitude: 4 V Pulse width: 90 μs Duration: 1, 6, 23 wk Double-blind study	MADRS; HDRS-24; FDG-PET Analyze the ratings for single items of both the HDRS-24 and MADRS scale to capture the aspects of anhedonia
2	Bewernick *et al.*, 2010 (50)	Germany	Acute antidepressant and antianhedonic effect of NAc DBS Dysfunction of the reward system	10	Anhedonia, depressive symptoms	NAc	Modality: bilateral Frequency: 100–150-Hz Amplitude: 2–4 V Pulse width: 60–210 μs Stimulation duration: in total 12 mo	HDRS-28; MADRS; AES
3	Bewernick *et al.*, 2012 (51)	Germany, United States	Anti-anhedonic and anxiolytic effects of NAc-DBS	11	Anhedonia, anxiety, depressive symptoms	NAc	Modality: bilateral Frequency: 130 Hz Amplitude: 2–4 V Pulse width: 90 μs Stimulation duration: 12 mo, 24 mo, 4 y	HDRS-28; MADRS; HAMA; SF-36; Neuropsychological assessment, the Hautzinger list of positive activities
4	Grubert *et al.*, 2011 (52)	Germany	Putative cognitive effects of NAc DBS	10	Attention, learning and memory, executive functions, visual perception, language	NAc	Modality: bilateral Frequency: 130 Hz Amplitude: 2–4 V Pulse width: 90 μs Stimulation duration: 12 mo	HDRS-28; MMSE; d2 attention burden test; VLMT; RVDLT HAWIE test; TMT; the Stroop Color and Word Test; VOT
5	Millet *et al.*, 2014 (53)	France	The NAc is a reward and pleasure center of the circuit involved in depression connected to the VTA, amygdala, hippocampus, orbitofrontal and medial prefrontal cortices, and motor territories of the caudate nucleus and globus pallidus.	4	Anhedonia	NAc	Modality: bilateral Frequency: 130 Hz Amplitude: 4–8 V Pulse width: 60 μs Stimulation duration: Up to 15 mo 1–5 mo NAc 5–9 mo for nonresponders DBS of caudate. NAc more promising than caudate, decrease in HDRS scores, none of the patients were either responders or remitters.	HDRS-17; GAF; CGI
6	Schlaepfer *et al.*, 2013 (55)	Germany	The slMFB as an interconnected center for the reward system	7	Motivation, antidepressant response	slMFB	Modality: bilateral Frequency: 130 Hz Amplitude: 2–3 V Pulse width: 60 μs Stimulation duration: mean stimulation current was 2.86 mA; stimulation initiated 1 wk after implantation. Twelve weeks for 7 patients, 4 patients tracked longer (up to 33 wk)	MADRS; AES HDRS-28; HAMA; SF-36; GAF
7	Coenen *et al.*, 2019 (54)	Germany	slMFB key function within the human reward system and its putative dysfunction in TRD	16 (15 TRD, 1 BP)	Antidepressant action	slMFB	Modality: bilateral Frequency: NS Amplitude: 2.1 mA, mean amplitude through whole 12 mo was 3.0 mA Pulse width: NS Stimulation duration: 12 mo	MADRS; HDRS-28; SF-36; GAF
8	Fenoy *et al.*, 2018 (56)	United States	slMFB as the center of the reward pathway connecting dopaminergic inputs from the VTA with the PFC	6	Antidepressant effect	slMFB	Modality: bilateral Frequency: 125–130 Hz Amplitude: 2–3 mA Pulse width: 60–75 μs Stimulation duration: After implantation, each patient was single blinded to stimulation onset for 4 wk, weekly assessments, in this 4-wk sham stimulation phase, each contact was explored to assess for threshold of side effects. By 4 wk poststimulation, each patient was unblinded to stimulation status and all received open-label active stimulation. Modalities: by Schlaepfer *et al.* (55)	HDRS-29; MADRS-20; GAF; HAMA; YMRS; CGI
9	Fenoy *et al.*, 2016 (57)	United States	slMFB as the center of the reward pathway connecting dopaminergic inputs from the VTA with the PFC	4	Motivational behaviors	slMFB	Modality: bilateral Frequency: 125–130 Hz Amplitude: 2–3 mA Pulse width: 60–75 μs Stimulation duration: In this interim analysis of an ongoing pilot study of 10 participants, weeks assessed the efficacy of MFB-DBS in a cohort of 4 patients with TRD over a 52-wk period using the MADRS as the primary assessment tool. Implanted patients entered a 4-wk single-blind sham stimulation period prior to stimulation initiation.	MADRS; HAMA; YMRS; CGI; HDRS-29; GAF
10	Bewernick *et al.*, 2018 (58)	Germany, United States	slMFB, key structure of the reward system, could have special influence on extraversion	30	Constructs of depression and personality are overlapping. Personality dimensions as predictors of the AD response. Two hypotheses: 1) State model assumes transient state-dependent changes of personality related to the severity of depression 2) Irreversible, long-term changes of personality after depressive episode, or scar model (depressed mood, cognitive deficits, lack of motivation)	slMFB	Modality: bilateral Frequency: 125–130 Hz Amplitude: 2–3 mA Pulse width: 60–75 μs Stimulation duration: 6 mo, 2 y, 5 y	HDRS-24; GAF; NEO-FFI
11	Kilian *et al.*, 2024 (59)	Germany	Reward and motivation-seeking behavior, antidepressant effects	12	Social skill improvement	slMFB	Modality: bilateral Frequency: NS Amplitude: NS Pulse width: NS Stimulation duration: 3 mo	BDI; MADRS; HDRS
12	Merkl *et al.*, 2013 (78)	Germany	The SCG, including BAs 25, 24, and 32, has shown abnormal metabolic activity in patients with depression.	6	Antidepressant effect, increased activity in the SCC, involved in mood regulation and self-generated sadness	SCG	Modality: bilateral Frequency: 130 Hz Amplitude: 2.5–10 V Pulse width: 90 μs Stimulation duration: 24 h, 24 wk, 36 wk	HDRS-24; MADRS; BDI
13	Kennedy *et al.*, 2011 (79)	Canada	Based on previous studies, report of extended follow-up after receiving DBS in SCG BA 25	20	NS	SCG	Modality: bilateral Parameters according to Mayberg *et.al.* (73) and Lozano *et al.* (72)	HDRS; SF-36
14	Conroy *et al.*, 2021 (68)	United States	Depressive disorders are structurally and functionally asymmetrical across hemispheres.	5	Antidepressant effect	SCC	Modality: unilateral 12 wk to each hemisphere, then bilateral 12 wk, frequency: 130 Hz Amplitude: 6–8 mA Pulse width: 90 μs Stimulation type: open-label DBS, monopolar Stimulation duration: 12 wk	HDRS-17; BDI-II High-resolution CT
15	Lozano *et al.*, 2008 (712)	United States, Canada	Depression is associated with increased activity in the SCG, direct modulation of SCG output	20	NS	SCG	Modality: bilateral Frequency: 130 Hz Amplitude: 3.5–5.0 V Pulse width: 90 μs Stimulation duration: 12 mo	HDRS-17 (mood, anxiety, sleep, somatic subscores); BAI; BDI; CGI-Severity; 18F-DG/15 O-water PET
16	Mayberg *et al.*, 2005 (88)	United States, Canada	The SC region (BA 25) is metabolically overactive in TRD. Disrupting focal pathological activity in limbic-cortical circuits using DBS can reverse symptoms in TRD.	6	Acute sadness, antidepressant effect	SCC	Modality: bilateral Frequency: 10–130 Hz Amplitude: 0.0–0.9 V Pulse width: 30–250 μs Stimulation duration: 1, 2, 3, 4, 5, 6 mo	HDRS-17; HDRS-24; MADRS; CGI; PANAS positive, negative scores; 18F-DG/15 O-water PET
17	Holtzheimer *et al.*, 2017 (73)	United States	In depression, BA 25 activity decreases in response to AD treatment among responders but remains unchanged in TRD. Structurally, BA 25 connects via monosynaptic pathways to brain regions involved in mood regulation, including the medial PFC, anterior cingulate gyri, hippocampus, amygdala, ventral striatum, thalamus, hypothalamus, and brainstem monoaminergic nuclei.	90 MDD	NS	SCC	Modality: bilateral Frequency: 130 Hz Amplitude: 4, 6, 8 mA Pulse width: 91 μs Stimulation type and duration: Randomized, double-blind, sham-controlled study Randomized to 6 mo of active or sham DBS, then 6 mo of open-label SCC DBS 2 wk after surgery: 1) stimulation group-immediate stimulation 2) 6 mo delayed stimulation (sham group)	MADRS; GAF; AES Secondary measures of efficacy: HDRS-17; IDS-C30; QIDS-SR; WSAS; PGI; CGI; QOL; HAMA
18	Holtzheimer *et al.*, 2012 (102)	United States	Based on DBS of SCC white matter was associated with antidepressant response Is DBS of the SCC effective for TRD in the context of MDD or BP?	10 MDD and 7 BP	NS	SCC	Modality: bilateral Frequency: 130 Hz Amplitude: 4–8 mA Pulse width: 90–91 μs Stimulation duration: 2 to 5 min of active stimulation at each contact. Single-blind sham stimulation for 4 wk followed by active stimulation for 24 wk, then they entered a single-blind discontinuation phase, stopped after the first 3 patients out of ethical concerns. Evaluated up to 2 y after onset of stimulation	HDRS-17; AES
19	Mclnerney *et al.*, 2017 (72)	Canada, United States, United Kingdom	Identify baseline cognitive predictors of treatment response to SCC DBS, compare neurocognitive performance prior to and 12 mo after DBS implantation	20 TRD	Cognitive predictors of treatment response	SCG	Modality: bilateral Frequency: 130 Hz Amplitude: 3.5–9 V Pulse width: 90 μs Stimulation duration: 12 mo	HDRS-17; Finger Tap-dominant Hand Test; Stroop test; COWA; HVLT; WCST
20	Eitan *et al.*, 2018 (74)	Israel, United States, France, United Kingdom	Protocol of optimization of stimulation in TRD of Cg25	9	Antidepressant-like effect	SCC, Cg25	Modality: bilateral Frequency: 130 vs. 20 Hz Amplitude: 3.5–9 V Pulse width: 90 μs Stimulation duration: 3–12 mo	MADRS; HDRS-17; QIDS-SR; Q-LES-Q; GAF; CGI; PGI
21	Merkl *et al.*, 2018 (60)	Germany, United States	Hyperactivity of SCG in TRD	8	Depression Idea to describe the results of delayed-onset design	SCG	Modality: bilateral Frequency: 130 Hz Amplitude: 2.5–10 V Pulse width: 90 μs Stimulation duration: 6, 12, 24 mo	HDRS-24
22	Riva-Posse *et al.*, 2018 (61)	United States	Functional hyperactivity of the SCG in MDD Test the potential utility of using individualized tractography map	11	Antidepressant effect	SCC	Modality: bilateral Frequency: 130 Hz Amplitude: 6 mA Pulse width: 91 μs Stimulation duration: 6 mo, 1 y	HDRS-17; GAF
23	Ramasubbu *et al.*, 2015 (62)	Canada	Effects of SCC DBS on serum BDNF	4	In the search for a biomarker of SCC-DBS antidepressant efficacy	SCC	Modality: bilateral Frequency: 130 Hz Amplitude: 3–5 V Pulse width: 90–450 μs Stimulation duration: 3, 6 mo	HDRS-17; BDNF
24	Bogod *et al.*, 2014 (63)	Canada	NS	4	Neuropsychological safety of SCG DBS	SCG	Modality: bilateral Frequency: 130 Hz Amplitude: 2.0–4.5 mA Pulse width: 91 μs Stimulation duration: 6 mo	HDRS-17; MADRS; IDS; NAART; WAIS-III; WMS-III; DKEFS; BNT; VFDT
25	Accolla *et al.*, 2016 (75)	Germany, Switzerland	Dysfunctional limbic circuits. The aim was to modulate the connections of the gyrus rectus and SCC (Cg25), BA 10, and mPFC.	5	Activation of networks to improve MDD symptoms	SCC, Cg25	Modality: bilateral Frequency: 130 Hz Amplitude: 5 V Pulse width: 90 μs Stimulation duration: 1, 3, 6, 9, 18, 24 mo	HDRS-24; BDI
26	Clark *et al.*, 2020 (104)	Canada	NS	16	Depressive symptoms	SCC	Modality: bilateral Frequency: 130 Hz Amplitude: 4–8 V Pulse width: 90–450 μs Stimulation duration: 12 mo	HDRS
27	Ramasubbu *et al.*, 2013 (65)	Canada	Optimization of SCC DBS	4	Effect between stimulus parameters and clinical effects in SCC-DBS treatment for TRD	SCC	Modality: bilateral Frequency: 2–185 Hz Amplitude: 0–10.5 V Pulse width: 60–450 μs Stimulation duration: 6 mo For an additional 6 mo after this double-blind period, all patients received open-label continuous stimulation using the stimulus parameters that were considered optimal at the end of the optimization phase.	HDRS-17; MADRS; HAMA; CGI; PANAS; VAS
28	Crowell *et al.*, 2019 (66)	United States	Response to MDD/BP SCC DBS	28	Long-term follow-up of SCC DBS, antidepressant response	SCC	Modality: monopolar, NS Frequency: 130 Hz Amplitude: 5–9 mA Pulse width: 87, 91 μs Stimulation duration: 4–8 y	HDRS; GAF
29	Elias *et al.*, 2022 (67)	Canada	Habenular involvement in response to SCC DBS	32	Mood and reward regulation	SCC	Modality: bilateral Frequency: 130 Hz Amplitude: individualized V Pulse width: 60–90 μs Stimulation duration: 12 mo	HDRS-17; volume measures of Hb and connectivity by fMRI
30	McCall *et al.*, 2020 (76)	United States	NS	12	Nonverbal behavior (react, engage/fidget, disengage)	SCC	Modality: bilateral Frequency: 130 Hz Amplitude: individualized V Pulse width: 60–90 μs Stimulation duration: 3–6 mo	BDI; HDRS-17; ethogram with 42 nonverbal behaviors
31	Aibar-Durán *et al.*, 2022 (103)	Spain	NS	17	Outcome analysis and correlation with lead position and electrical parameters determine whether the lead position within the SCG or the electric parameters could be related to response to the therapy.	SCG	Modality: bilateral, 13 patients received bipolar stimulation/4 monopolar Frequency: 130–135 Hz Amplitude: 4–5 V Pulse width: 120–210 μs Stimulation duration: 6 mo, 24 mo, 5 y	HDRS-17
32	Clark *et al.*, 2020 (104)	Canada, United States	Previous encouraging results of the SCC’s central position in overlapping white matter circuits implicated in mood regulation Different SCC region activity, tract-based analysis	19	Depressed mood	SCC	Modality: bilateral Medtronic 3387 unipolar Frequency: 130 Hz Amplitude: 4–8 V Pulse width: 90–450 μs Stimulation duration: 6, 12 mo	HDRS-17
33	Conen *et al.*, 2018 (105)	United Kingdom	Cg25 metabolism is increased in MDD, and remission is associated with normalization of this hypermetabolism. Aim to modulate dysfunctional neuronal networks	8	Depressed mood, antidepressant effect	SCC Cg 24,25 VAC, NAc	Modality: bilateral Medtronic 3387 2 electrodes with all 4 contacts in Cg24,25 2 electrodes in the VAC, NAc Frequency: NS Amplitude: NS Pulse width: NS Stimulation duration: 16–45 mo	MADRS; HDRS-17; PET
34	Williams *et al.*, 2016 (47)	United States, Lebanon	Hypoactivity of the left DLPFC, hyperactivity of the right DLPFC; model used in rTMS FPC and especially BA 10—node in mood-regulatory circuitry, increased resting-state activity in patients with depression	5	Depressive symptoms	EpCS of the FPC and DLPFC	Modality: bilateral Frequency: 60–130 Hz Amplitude: 4.5–6.5 V Pulse width: 210 μs Stimulation duration: 7 mo, 1 y, 2 y, 5 y	HDRS-24
35	Nahas *et al.*, 2010 (46)	United States	The anterior and midlateral prefrontal cortices play complementary roles in integrating emotional and cognitive experiences and in modulating subcortical regions.	5	Mood regulation (particularly depressed mood), cognitive-emotional integration	EpCS of AFPMPC	Modality: bilateral Frequency: 60 Hz Amplitude: 2–4 V Pulse width: NS Stimulation duration: 2 wk adjusting, then 17 wk	HDRS-24; MADRS; IDS-SR
36	Zhang *et al.*, 2022 (21)	China, United Kingdom	Dysfunction of the reward system in depression	7	Depressive symptoms in patients with TRD or bipolar disorder, mood	Bilateral habenula	Modality: bilateral Frequency: NS Amplitude: NS Pulse width: NS Stimulation duration: NS	HDRS-21; YMRS; HAMA; PSQI; health status; functional impairment; quality of life FU; SF-36
37	Fitzgerald *et al.*, 2018 (77)	Australia, Canada	BNST role in the regulation of mood and anxiety	5	Mood, anxiety	BNST	Modality: bilateral Frequency: 60–130 Hz Amplitude: 4.5–6.5 V Pulse width: 210 μs Stimulation duration: 8 wk	MADRS; BDI (II); QOL; HDRS-17
38	Malone *et al.*, 2010 (106)	United States	Chronic stimulation of the VC/VS improve function and mood in patients with OCD. Significantly less VS response to positive stimuli in patients with depression compared with control participants. SC region metabolically hyperactive in MDD	Additional 2 patients in total 17 for this study (first one published in 2009)	Depressive mood, anxiety	VC/VS	Modality: bilateral Frequency: 100–130 Hz Amplitude: 2.5–8 V Pulse width: NS Stimulation duration: 3, 6, 12 mo, last follow-up average 37.4 (range 14–67) mo	HDRS; MADRS; GAF
39	Malone *et al.*, 2009 (107)	United States	Chronic stimulation of the VC/VS improves function and mood in patients with OCD. Significantly less VS response to positive stimuli in patients with depression compared with control participants. SC region metabolically hyperactive in MDD	15	Depressive mood, anxiety	VC/VS	Modality: bilateral Frequency: 100–130 Hz Amplitude: 6.7–8.4 V Pulse width: 90–210 μs Stimulation duration: All patients received continuous stimulation and were followed for a minimum of 6 mo to longer than 4 y.	MADRS; HDRS-24; SAEs; GAF
40	Dougherty *et al.*, 2015 (108)	United States	Marked improvement in comorbid depressive symptoms when using DBS of the VC/VS for patients with OCD. Overlap of circuitry implicated in the pathology of MDD and OCD	30	Depressive symptoms	VC/VS	Modality: bilateral Frequency: NS Amplitude: 8 V Pulse width: 90–210 μs Stimulation duration: All patients received continuous stimulation and were followed for a minimum of 4 wk to 3 y.	MADRS
41	Kubu *et al.*, 2017 (109)	United States	Observation of cognitive parameters and changes in depression, evaluation of cognitive outcome after VC/VS DBS	25	NS	VC/VS	Modality: bilateral Frequency: NS Amplitude: 8 V Pulse width: 90–210 μs Stimulation duration: 16 wk	MADRS
42	Hitti *et al.*, 2021 (110)	United States	Dysfunctional brain networks	8	NS	VC/VS	Modality: bilateral 3391 Medtronic Bipolar more than monopolar Frequency: 130 Hz Amplitude: 7.2 V (SD = 2.6) Pulse width: 90–210 μs Stimulation duration: Max 11 (SD = 0.4) y	MADRS
43	Bergfeld *et al.*, 2016 (111)	Netherlands	Strong antidepressive effect identified in patients with OCD following active NAc and vALIC DBS	25	Depressive symptoms	vALIC	Modality: bilateral Frequency: 130 or 180 Hz Amplitude: 2.5–6.0 V Pulse width: 90 μs Stimulation duration: Four weeks, after a maximum of 52 wk	HDRS-17; MADRS; IDS-SR; IDS-SF
44	Raymaekers *et al.*, 2017 (95)	Belgium	DBS of BNST in immediate vicinity of the VC/VS and NAc. BNST part of limbic system with projections to the MFB, NAc, possible relay center of the processing of reward, stress, and anxiety	7	Comorbid depressive symptoms	ITP, IC, BNST	Modality: bilateral Frequency: NS Amplitude: NS Pulse width: NS Stimulation duration: stimulation initiated 2–4 wk after implantation, 5 mo optimization of parameters, IC/BNST and ITP ON/OFF, randomized crossover. In total 6 mo (3 × 2)	HDRS-17
45	Van der Wal *et al.*, 2020 (49)	Netherlands	NS	25	Depressive symptoms, DBS in patients with TRD. Assess the efficacy and safety of DBS targeted at the vALIC	vALIC	Modality: bilateral Frequency, amplitude, Pulse width (μs): as Bergfeld *et al.* (90) Stimulation duration: 1 y maintenance, 1 y optimization	HDRS-17; MADRS; IDS-SR
46	Bergfeld *et al.*, 2022 (48)	the Netherlands	RCTs showed the superiority of active DBS vALIC compared with sham BNST, MFB	25	NS	vALIC	Modality: bilateral Frequency: 130 or 180 Hz Amplitude: 2.5–6.0 V Pulse width: 90 μs Stimulation duration: 6 y	HDRS-17 Response defined as ≥50% reduction of HDRS score in the baseline WHOQOL-BREF
47	Ramasubbu *et al.*, 2020 (112)	Canada	NS	22	NS Aim to assess the efficacy and tolerability of LPW vs. SPW DBS SCC (higher amplitudes and longer pulse width stimulation increase the spatial distribution of the electrical field and volume of tissue activated around the electrode)	SCC	Modality: bilateral Frequency: 130 Hz Amplitude: 2.5–6.0 V Pulse width: 90 (SPW) or 210–450 (LPW) μs Stimulation duration: 6 mo	HDRS-17
48	Wang *et al.*, 2024 (45)	China	Hypothesis that combined stimulation may synergistically exert the therapeutic effect, the BNST and NAc are anatomically interconnected by dense fiber tracts and are reported to share a cooperative functionality.	23	Improvement of depressive symptoms	BNST + NAc	Modality: bilateral Frequency: 130 Hz Amplitude: 2.0–6.0 V Pulse width: 90 μs Stimulation duration: 4–24 mo	HDRS-17; MADRS; HAMA-14; DSSS

AD, antidepressant; AES, Apathy Evaluation Scale; AFPMPC, anterior frontal poles and midlateral prefrontal cortex; BA, Brodmann area; BDI, Beck Depression Inventory; BNST, bed nucleus of the stria terminalis; BNT, Boston Naming Test; BP, bipolar II disorder; Cg25, anterior cingulate cortex BA 25; CGI, Clinical Global Impressions Scale; COWA, Controlled Oral Word Association Test; DBS, deep brain stimulation; D-KEFS, Delis-Kaplan Executive Function System; DLPFC, dorsolateral prefrontal cortex; DSSS, Depression and Somatic Symptoms Scale; EpCS, epidural prefrontal cortical stimulation; FDG-PET, fluorodeoxyglucose positron emission tomography; FPC, frontopolar cortex; GAF, Global Assessment of Functioning Scale; HAMA, Hamilton Anxiety Rating Scale; HAWIE, Hamburg Wechsler Intelligence Test for Adults; HDRS-17/24/28, 17/24/28-item Hamilton Depression Rating Scale; HVLT, Hopkins Verbal Learning Test; IC, internal capsule; IDS-SR, Inventory of Depressive Symptomatology Self Report; ITP, inferior thalamic peduncle; LPW, long pulse width; MADRS, Montgomery–Åsberg Depression Rating Scale; MD, major depression; MDD, major depressive disorder; MFB, medial forebrain bundle; NAc, nucleus accumbens; NAART, North American Adult Reading Test; NEO-FFI, Test of Five Factor Theory of Personality; NS, not specified; OCD, obsessive-compulsive disorder; Q-LES-Q, Quality of Life and Satisfaction Questionnaire; QIDS-SR, Quick Inventory of Depressive Symptomatology Self-Report; QOL, Quality of Life Scale; PANAS, Positive and Negative Affect Schedule; PGI, Patient Global Impressions scale; RVDLT, Rey Visual Design Learning Test; rTMS, repetitive transcranial magnetic stimulation; PSQI, Pittsburgh Sleep Quality Index; SAEs, serious adverse events; SCC, subcallosal cingulate cortex; SCG, subgenual cingulate gyrus; SF-36, 36-item Short Form Survey; Sg25, subgenual cingulate BA 25; slMFB, superolateral medial forebrain bundle; SPW, short pulse width; TMT, Trail Making Test; TRD, treatment-resistant depression; vALIC, ventral anterior limb of the internal capsule; VC, ventral internal capsule; VFDT, Benton Visual Form Discrimination Test; VLMT, Verbal Learning and Memory Test; VOT, Visual Organization Test; VS, ventral striatum; VTA, ventral tegmental area; WAIS, Wechsler Adult Intelligence Scale; WCST, Wisconsin Card Sort Test; WHOQOL-BREF, World Health Organization Quality-of-Life Scale; WMS, Wechsler Memory Scale; WSAS, Work and Social Adjustment Scale; YMRS, Young Mania Rating Scale.

## Results

**Figure 1:**
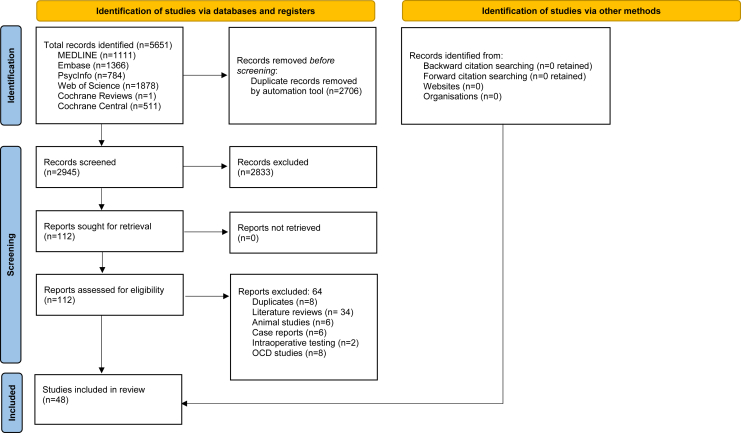
Preferred Reporting Items for Systematic Reviews and Meta-Analyses (PRISMA) 2020 flow diagram. OCD, obsessive-compulsive disorder.

The initial search yielded 5651 titles. After removing duplicates, 2945 unique citations remained. Following title and abstract screening, 2833 records were excluded. Of the 112 full-text studies assessed for eligibility, 66 were excluded, and all full texts were successfully retrieved (0 reports not retrieved). Two authors (LP and JE) independently screened and extracted data from the 48 peer-reviewed studies (Tables 1 and 2) that met inclusion criteria (Figure 1).

**Figure 2:**
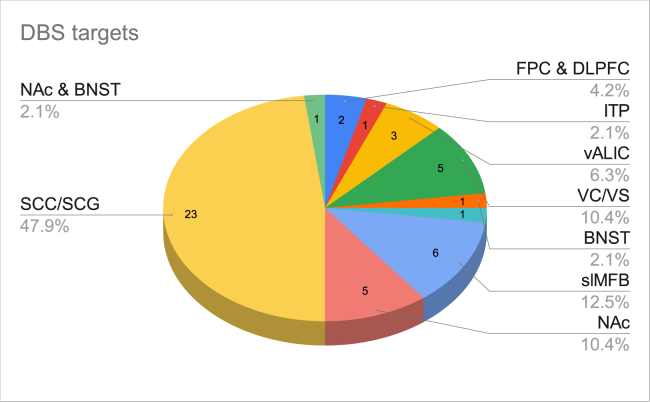
Deep brain stimulation (DBS) targets. BNST, bed nucleus of the stria terminalis; DLPFC, dorsolateral prefrontal cortex; FPC, frontopolar cortex; ITP, inferior thalamic peduncle; NAc, nucleus accumbens; SCC, subcallosal cingulate cortex; SCG, subgenual cingulate gyrus; slMFB, superolateral medial forebrain bundle; vALIC, ventral anterior limb of the internal capsule; VC, ventral capsule; VS, ventral striatum.

The selection criteria for TRD are detailed in Table 1. Key study characteristics, including the origin of the studies, the conceptual frameworks of depression, targeted symptoms, DBS parameters, and outcome measures, are summarized in Table 2, alongside data relevant to the review’s core questions and objectives.

### Definition of TRD

Regarding the definition of TRD, we identified several criteria such as response to pharmacotherapy, lifetime MDD episodes, the duration of the current depressive episode, whether ECT or evidence-based psychotherapy had been attempted, the presence of other psychiatric comorbidities, and the use of psychotropic medication at the time of DBS.

Data regarding response to pharmacotherapy exhibited high variability. Lifetime MDD episodes ranged from 2 to 20, with 13 studies not specifying this aspect (28%). The duration of the current depressive episode ranged from 1 to 10 years, with 4 studies omitting this information (9%). Only 2 studies did not specify whether patients underwent ECT (4%), while the remaining studies (96%) detailed the selection of patients for DBS, with a diverse range of ECT treatment intensities and frequencies; 41 (85%) reported the use of evidence-based psychotherapy as a therapeutic intervention for patients selected for DBS, although the duration of therapy varied significantly across studies. Comorbidities were reported in 10 studies (21%) and were absent in 32 studies (67%), while 6 studies did not specify this aspect (12%). Current psychotropic medication was continued for patients in 33 studies (68%), while 15 studies (32%) did not specify whether patients were still receiving pharmacological treatment. Patients with TRD included in the current review had a history of multiple failed antidepressant (AD) trials, often involving ≥3 distinct classes of medication. On average, individuals had undergone between 7 and >20 unsuccessful pharmacological treatments, reflecting a high level of resistance to conventional therapies. Most studies included participants who failed at least 4 to 5 AD trials, frequently across different classes and in combination with various augmentation strategies. These patients were often prescribed multiple concurrent psychotropic medications (typically ranging from 3 to ≥6), which may include selective serotonin reuptake inhibitors, serotonin-norepinephrine reuptake inhibitors, atypical antipsychotics, benzodiazepines, mood stabilizers, and in some cases, monoamine oxidase inhibitors or ketamine. Augmentation treatments such as lithium, atypical antipsychotics, mood stabilizers, and thyroid hormones are commonly part of their therapeutic history. At the time of study entry, patients were typically maintained on a stable medication regimen, often involving complex combinations of ADs, antipsychotics, lithium, benzodiazepines, mood stabilizers, thyroid hormones, and other agents.

### Anatomical DBS Targets and Conceptual Frameworks of Depression

Among 48 studies, 46 used intracranial bilateral DBS. The anatomical targets chosen for stimulation were the subcallosal cingulate cortex/subgenual cingulate gyrus (SCC/SCG) (*n* = 23), superolateral MFB (slMFB) (*n* = 6), nucleus accumbens (NAc) (*n* = 5), VC/VS (*n* = 5), ventral anterior limb of the internal capsule (vALIC) (*n* = 3), Hb bilaterally (*n* = 1), bed nucleus of the stria terminalis (BNST) (*n* = 1), ITP (*n* = 1), and the internal capsule and the BNST (*n* = 1). Only one study used combined BNST and NAc DBS (*n* = 1) (Figure 2).

Among the 48 studies reviewed, 20 studies (41.7%) targeted the normalization of negative affect, 8 studies (16.7%) focused on anhedonia; 12 studies (25%) were classified under other targets, including cognition, biomarkers, and related domains; and 8 studies (16.7%) did not specify any target symptom.

Only one study used 2-target combined stimulation (45). Two studies used bilateral epidural cortical stimulation, one of the frontopolar cortex (FPC) and the left dorsolateral prefrontal cortex (DLPFC) and the other of the anterior and midlateral PFC. This selection relied on the fact that the anterior and midlateral prefrontal cortices play a role in integrating emotional and cognitive experiences and modulating subcortical regions (46). Additionally, Williams *et al.* (47) suggested that a hyperactive right DLPFC and a hypoactive left DLPFC, along with the FPC, are key nodes in the mood regulation circuitry.

Various conceptual frameworks were discussed across the studies, including clinical models [e.g., mood and anxiety regulation through the BNST, antidepressant effects mediated via the vALIC (48,49)], circuit- and network-based neurobiological models [e.g., dysfunctional reward systems involving the NAc and the slMFB (21,50, 51, 52, 53, 54, 55, 56, 57, 58, 59)], and neurofunctional models [e.g., increased metabolism in the SCG region and hypoactivity of the DLPFC (46,47,60, 61, 62, 63, 64, 65, 66, 67)].

Given the heterogeneity of the included studies, ranging from clinical trials to mechanistic investigations, obtaining relevant information on the specific clinical or symptomatic targets of depression proved challenging. In many cases, this parameter appeared to receive less emphasis than aspects such as patient selection criteria or DBS parameters. Eight studies did not explicitly describe the depressive symptoms that they aimed to target; of these, 4 studies focused on SCC/SCG, 2 focused on VC/VS, and 2 focused on vALIC. Among the studies that did reference clinical features, common targets included core depressive symptoms such as anhedonia, low mood (sadness), and motivational deficits, as well as broader references to antidepressant effects. However, these symptoms were often mentioned without being explicitly linked to a structured clinical framework or a clearly defined symptom cluster of TRD. In parallel, several studies framed their rationale using neurobiological constructs (e.g., dysfunction of reward or affective networks), without specifying how these related to individual symptom dimensions. This highlights a lack of consistency in how symptomatology is conceptualized and reported across the DBS literature in TRD.

### DBS Parameters

**Figure 3:**
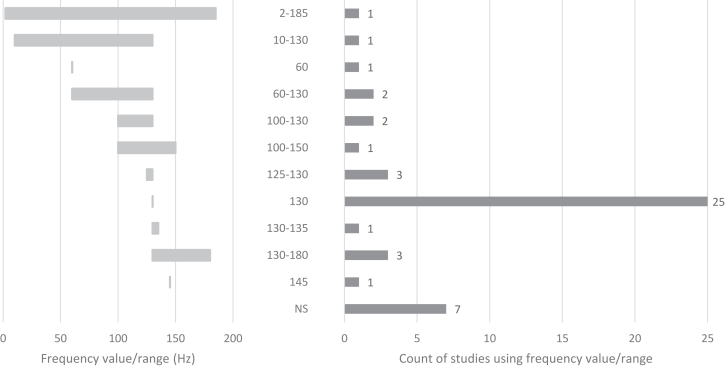
Frequency/value range indicated in Hz. Number of studies using frequency value range (before indicated as count of studies).

**Figure 4:**
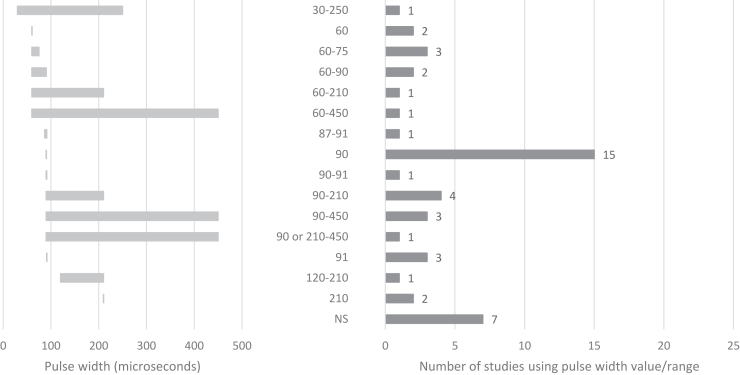
Pulse width distribution and number of studies using the pulse width.

**Figure 5:**
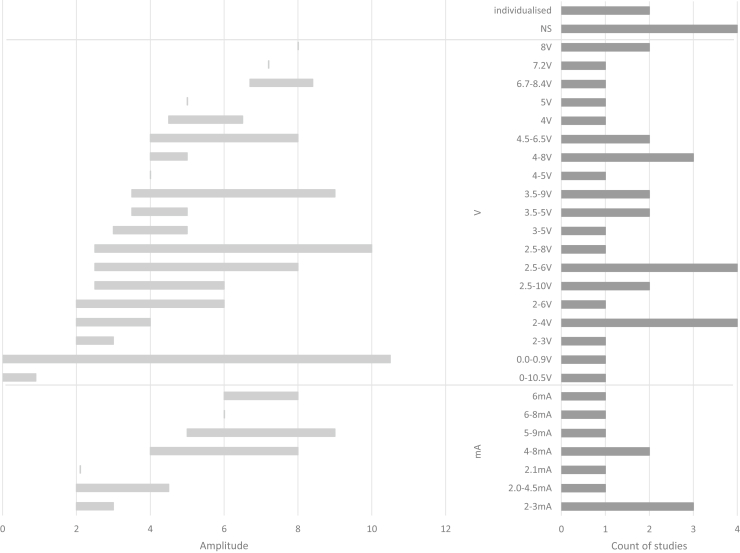
Number of studies and amplitude ranges. Amplitude is reported in volts (V) or milliamperes (mA).

**Figure 6:**
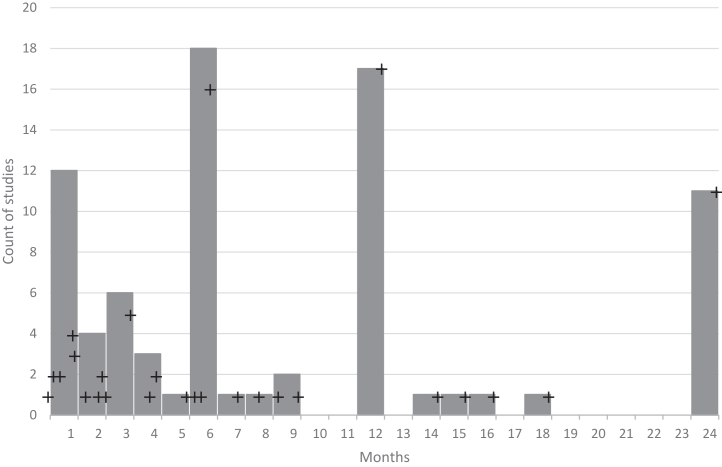
Number of unique studies by duration in months for 0 to 2 years. Vertical bars represent the total number of studies falling in that month (e.g., 24 hours; 1, 2, and 4, weeks; and 1 month for the period 0–1 month). Black crosses represent the unique durations as reference in the studies (e.g., 8 weeks and 2 months are distinct durations) on a real-time horizontal axis. The number of studies for the duration is on the vertical axis; for studies with more than 1 duration, it is included for each distinct period.

**Figure 7:**
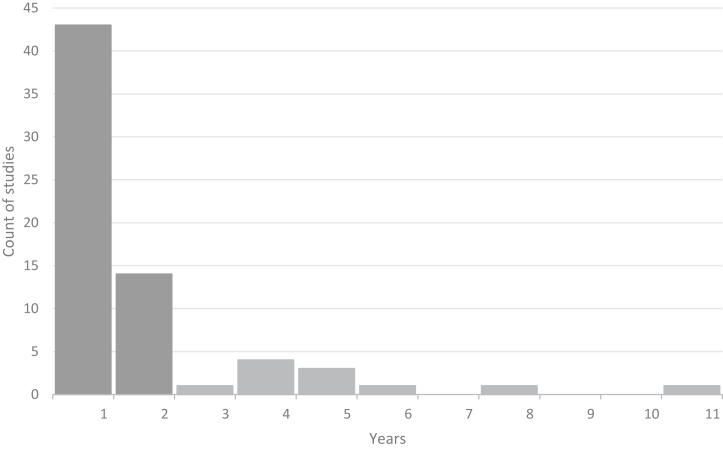
Number of unique studies by duration in years. For studies that have multiple durations spanning several years (e.g., 6, 12, 24 months), the study was not counted twice for year 1; instead, it was counted once each for year 1 and year 2.

DBS parameters, modalities, and durations varied across studies, although most provided clear descriptions. Nearly all studies used bilateral DBS, with only one failing to specify the modality. Some distinguished between monopolar and bipolar contacts, while Conroy *et al.* (68) uniquely began with unilateral DBS before transitioning to bilateral DBS. The most common frequency was 130 Hz, although 5 studies (11%) explored lower frequencies (10–60 Hz), and 1 investigated a range of 100 to 150 Hz. Seven studies (14%) did not specify frequency, and rationales for frequency selection were rarely detailed. Amplitudes were reported in volts (71%) or milliamperes (20%), with ranges of 2.5 to 10 V or 2 to 9 mA, while pulse widths, typically in microseconds, varied widely, from 30 to 450 μs. Stimulation durations spanned 1 week to 11 years. An overview of the stimulation parameters is provided in Figure 3, Figure 4, Figure 5, Figure 6, detailing frequency range (Figure 3), pulse width (Figure 4), amplitude (Figure 5), and DBS duration (Figures 6 and 7).

### Outcome Measures

Primary outcomes were well defined, with HDRS and MADRS being used most often to measure DBS efficacy in TRD. A 50% or greater reduction in baseline scores signified a response. Two studies (4%) exclusively used MADRS, while most (96%) utilized HDRS alone or with other tools such as MADRS, Global Assessment of Functioning (GAF) (69), and Hamilton Anxiety Rating Scale (70). Secondary outcomes varied significantly. Five studies (11%) used the Inventory of Depressive Symptoms—Self-Report, and 6 (13%) included the Clinical Global Impressions Scale. Only 2 studies (4%) analyzed 17-item HDRS subscores, focusing on mood, anxiety, sleep, and somatic symptoms (21,71). Zhang *et al.* (21) evaluated mania, sleep, and anxiety items. A diverse array of secondary measures was used to assess facets of depression. The Wisconsin Card Sorting Test (WCST) (72) was used in 1 study (2%), while the Patient Global Impressions Scale (PGI) appeared in 2 studies (4%) (73,74). Six studies (13%) used the Beck Depression Inventory (BDI) (68,71,75, 76, 77, 78), and 1 study (2%) used the Work and Social Adjustment Scale (WSAS) (73). Five studies (10%) incorporated the 36-item Short Form Survey (21,51,54,55,79), while the Quality of Life Scale (QOL) appeared in 2 studies (4%) (73,77). Additionally, 1 study each used the World Health Organization Quality-of-Life Scale-Brief version (WHOQOL-BREF) (48) and the Depression and Somatic Symptoms Scale (45). These measures underscore the broad variability in evaluating DBS outcomes and highlight the need for more standardized tools.

## Discussion

Unlike prior reviews focused mainly on treatment outcomes or target-specific results, in this scoping review, we integrated both clinical and mechanistic studies to offer a broader, multidimensional perspective on DBS in TRD. We introduced a symptom-targeted framework aligned with precision psychiatry but still underused in the DBS field. By systematically analyzing stimulation parameters, we identified key knowledge gaps and advocate for viewing depression as a networkopathy, supporting more personalized, circuit-informed neuromodulation strategies. Our findings highlight 1) significant variability in TRD definitions; 2) inconsistent application of conceptual frameworks, differing by target, parameters, and duration; and 3) limited attention to the heterogeneity of depressive symptoms, with the diverse phenomenology of TRD rarely being addressed in a structured manner.

The standard definition of TRD—failure to respond to at least 2 adequate AD regimens—has frequently been applied inconsistently in DBS research (80). This variability in definitions, along with differences in prior treatments, illness duration, psychiatric comorbidities, and concurrent medications, complicates the comparison of patient response across studies and undermines the generalizability of findings. Systematic reviews have highlighted this heterogeneity as a key limitation of TRD research (18,81). Moreover, the commonly accepted TRD definition often overlooks critical clinical factors, such as psychiatric and somatic comorbidities, which are highly prevalent in TRD populations [83% and 70%, respectively, in a recent study (82)]. To better reflect the complexity of TRD, broader diagnostic criteria and sociodemographic data should be routinely included in study protocols. Achieving greater consistency in DBS research requires the adoption of standardized diagnostic and reporting frameworks (83). We recommend a minimum reporting set that includes diagnostic criteria, depression subtype, illness course, duration of current and past episodes, chronicity, number and classes of failed treatments, adequacy and duration of each trial, augmentation strategies, psychotherapy history, and prior somatic interventions (e.g., ECT, transcranial magnetic stimulation [TMS]). This approach does not necessitate a strict consensus on a single TRD definition, but it would significantly improve transparency, reproducibility, and the interpretability of DBS trials. By encouraging the scientific community to implement and adhere to such standards, future studies will be better positioned to capture the full clinical picture of TRD and evaluate DBS outcomes in a more rigorous and comparable manner.

Our review demonstrates considerable variability in the conceptualization of depression across studies, including clinical dimension–based, circuit-based, network-based, or neurofunctional models (3,20,23,84, 85, 86, 87, 88, 89, 90). Most studies adopted a symptom-based approach by targeting brain circuits associated with MDD’s core criteria: negative affect and anhedonia. In these studies, negative affect is addressed through areas such as the SCC and LHb, while anhedonia is linked to regions such as the vALIC, VC/VS, ITP, MFB, NAc, and BNST (23). Notably, 26 studies (57%) in this review focus on the SCC, a target grounded in Mayberg’s model of depression, which views SCC as a regulatory hub for frontal cortical and limbic activity (3). This theory suggests that stabilizing SCC function, acting as a homeostatic fulcrum, could normalize disrupted activity between limbic and frontal areas (84, 85, 86). However, studies have often used SCC and SCG interchangeably without clarification. The SCG, located in Brodmann area 25, is a critical mood regulation region within the SCC capable of modulating activity in frontal and limbic areas (85). Hypermetabolism in the SCG is strongly linked to TRD, with high-frequency DBS aimed at reversing this hyperactivity (87). Moreover, this symptom-specific focus underscores a divide in how depression is conceptualized but fails to offer a comprehensive, personalized treatment approach. Depression’s impact extends beyond emotional and reward systems to include cognitive domains such as attention, memory, decision making, and psychomotor activity.

Two studies of the review showed reduced activity in the left DLPFC and dorsal ACC, together with increased right ACC activity (46,47), consistent with the interhemispheric imbalance model of depression, where a hyperactive right hemisphere coexists with hypoactivity and reduced metabolism on the left (88, 89, 90). Conroy *et al.* (68) and Guinjoan *et al.* (91) explored unilateral DBS targeting the SCC, with Conroy demonstrating that left-sided stimulation significantly reduced depression scores, unlike right-sided stimulation, thereby highlighting the potential for personalized therapy. This model supports repetitive TMS in MDD treatment, using high-frequency stimulation of left Brodmann areas 4, 9, and 46 and low-frequency stimulation on the right (92,93).

A dysfunctional reward system is often seen as a core feature of MDD, with the NAc playing a central role (20). The slMFB also plays a key role, connecting major components of the reward system, including the NAc, ventral tegmental area (VTA), ventromedial and lateral nuclei of the hypothalamus, and the amygdala (21,50,53, 54, 55, 56, 57, 58, 59,67,94,95). The hypothesis that modulating the reward system near the VTA could effectively reduce depression symptoms has been supported by studies on bilateral DBS of the slMFB (55,59,96). This system, often referred to as the limbic circuit, is closely linked to the cognitive circuit, which involves the central striatum and DLPFC (87).

The absence of a consensus on TRD definitions, coupled with the lack of a truly unified model of depression—which remains more aspirational than actual—alongside limited investigation into its multidimensional phenomenology, highlight critical gaps in current DBS research. This complexity is further compounded by the variability in stimulation targets, highlighting the necessity to move beyond a singular conceptualization of depression. Studies should clearly define the symptom clusters being targeted, justify target selection based on neurobiological rationale, and report patient profiles to reflect clinical heterogeneity (e.g., melancholic vs. atypical) (7, 8, 9, 10). A more precise, symptom-based approach could enhance the interpretability, reproducibility, and ultimately the effectiveness of DBS interventions for TRD.

There is significant variability in DBS parameters for TRD, including amplitude, frequency, pulse width, and stimulation duration, which hinders comparability across studies. Although bilateral DBS is commonly used, the heterogeneity in other technical aspects reflects differing trial designs and conceptual depression frameworks. Most studies have used stimulation settings (100–130 Hz frequency) adapted from Parkinson's disease protocols, despite fundamental differences in pathophysiology (24). Currently, DBS operates through open-loop systems, with fixed stimulation, adjusted based on clinical response. However, closed-loop systems, which adapt stimulation in real time based on neural activity, represent a promising direction for TRD treatment, as has been seen in movement disorders, epilepsy, and pain management (17,97,98). These advancements could enhance the personalization and effectiveness of DBS in TRD.

In studies of DBS for TRD, primary outcomes have typically been based on MADRS and HDRS scores, with response defined as a >50% reduction from baseline. However, this focus often neglects essential aspects such as quality of life, functional capacity, and patient-reported well-being. While open-label studies have reported high response rates (up to 90%) (17,24), randomized controlled trials have yielded less robust outcomes (99,100), likely due to confounding factors and the limitations of current rating scales. MADRS omits several DSM-5 MDD criteria, and HDRS disproportionately emphasizes somatic symptoms (23), limiting the utility of these measures for capturing TRD’s complexity. Improving quality of life should be prioritized over reducing isolated symptoms. Rabin *et al.* (17) called for broader evaluation tools that capture diverse depression features. Some studies now include secondary outcomes such as the GAF, WCST, PGI, BDI, WSAS, QOL, and WHOQOL-BREF, but only 2 research groups have analyzed subscores to track specific symptom changes (71,94). We propose a framework distinguishing 3 levels of remission: symptomatic, syndromic, and functional. Symptomatic recovery refers to the reduction of clinical symptoms, and syndromic recovery involves remission of the full diagnostic syndrome, while functional recovery focuses on the restoration of daily living and social roles. Functional remission is the key, as many patients may be symptom-free but still function poorly. However, it remains difficult to assess. The GAF fails to clearly separate symptom severity from functional impairment. In contrast, psychometrically robust tools such as the WHO Disability Assessment Schedule (WHODAS) 2.0, Self-Directed Search (SDS), and WSAS provide greater specificity and conceptual clarity for assessing functional outcomes in DBS research.

Current limitations in evaluation tools may obscure DBS’s true effectiveness. Subscore analysis and improved rating scales such as the Snaith-Hamilton Pleasure Scale for anhedonia are crucial for tracking outcomes linked to specific symptom changes and enhancing DBS efficacy assessment.

## Limitations

We excluded gray literature to prioritize studies with well-defined methodologies and detailed reporting, thereby ensuring greater reproducibility and transparency. Gray literature often lacks methodological rigor and may include outdated or incomplete data, increasing the risk of unreliable findings. However, the small sample size of studies in this scoping review limits the generalizability of results. In DBS for TRD, individual responses vary widely based on patient characteristics, stimulation parameters, and targeted regions. Smaller samples may not represent the broader population, reducing the applicability of conclusions to real-world clinical settings.

## Conclusions

DBS holds promise as a treatment for TRD, particularly given the global burden of depression and the limitations of current therapies (101). Despite heterogeneity in study designs, this diversity highlights the growing interest in the field. Future research should focus on optimizing designs, standardizing inclusion criteria, and exploring bilateral and multitarget DBS approaches. Common scales such as MADRS and HDRS may not fully capture DBS outcomes, especially regarding quality of life and patient-reported measures. As MDD and TRD are increasingly understood as network-level disorders, refining symptom-based targeting and developing personalized interventions are crucial. Recovery should be evaluated not only by symptom reduction but also by functional outcomes, emphasizing restored daily functioning and well-being. However, the interconnectivity of neural circuits complicates direct symptom-to-target mapping, requiring more nuanced brain-behavior models. Beyond therapy, DBS remains a valuable research tool for exploring depression neurobiology. A more integrated, data-driven approach will be key to advancing DBS as an effective intervention.
