# Supplementary material for: Has Deep Brain Stimulation Shown Its Full Potential in Treatment-Resistant Depression? A Scoping Review
Source: Biol Psychiatry Glob Open Sci. 2025 Dec 23;6(3):100682. doi: 10.1016/j.bpsgos.2025.100682 (PMC12925169; doi:10.1016/j.bpsgos.2025.100682)
Supplement: Supplemental Text [file mmc1.pdf]

SUPPLEMENTARY INFORMATION

Has Deep Brain Stimulation Shown Its Full Potential in Treatment-Resistant Depression? A Scoping Review

Puke *et al.*

Bibliographic database search strategies

Joëlle Rosselet Amoussou, Medical Library-Cery, Lausanne University Hospital and University of Lausanne, Site de Cery, 1008 Prilly, Switzerland, ORCID 0000-0001-6871-5350

The research strategies were peer reviewed by another information specialist prior to execution.

Embase.com

1366 results on 24 September 2024

((('brain depth stimulation'/mj OR "deep brain stimul*":ab,ti,kw) AND ('treatment resistant depression'/mj OR depress*:ti OR (resistant NEXT/2 depress*):ab,ti,kw)) OR (('brain depth stimulation'/exp OR 'deep brain stimulator'/exp OR 'transcranial magnetic stimulation'/exp OR (("deep brain" NEXT/3 stimul*) OR DBS OR "brain depth stimul*" OR "intracranial brain stimul*" OR "transcranial magnetic stimul*" OR TMS OR rTMS OR "theta burst stimul*" OR TBS):ab,ti,kw) AND (multitarget* OR ((multiple OR multi OR dual OR combined OR several) NEXT/3 (target* OR stimulation)) OR (bilateral NEXT/6 stimul*) OR (bilateral NEXT/3 (DBS OR TBS OR rTMS OR EpCS OR implentation*)) OR "ventral capsule/ventral striatum"):ab,ti,kw AND ('depression'/exp OR 'mood disorder'/de OR (depression* OR depressive OR bipolar OR "mood disorder*" OR "affective disorder*"):ab,ti,kw))) NOT ([animals]/lim NOT [humans]/lim) NOT ([conference abstract]/lim OR [conference paper]/lim)

Medline ALL Ovid

Ovid MEDLINE(R) ALL 1946 to September 23, 2024
1111 results on 24 September 2024

(((*Deep Brain Stimulation/ OR "deep brain stimul*".ab,ti,kf.) AND (*Depressive Disorder, Treatment-Resistant/ OR depress*.ti. OR (resistant ADJ2 depress*).ab,ti,kf.)) OR ((Deep Brain Stimulation/ OR Transcranial Magnetic Stimulation/ OR (("deep brain" ADJ3 stimul*) OR DBS OR "brain depth stimul*" OR "intracranial brain stimul*" OR "transcranial magnetic stimul*" OR TMS OR rTMS OR "theta burst stimul*" OR TBS).ab,ti,kf.) AND (multitarget* OR ((multiple OR multi OR dual OR combined OR several) ADJ3 (target* OR stimulation)) OR (bilateral ADJ6 stimul*) OR (bilateral ADJ3 (DBS OR TBS OR rTMS OR EpCS OR implentation*)) OR "ventral capsule/ventral striatum").ab,ti,kf. AND (Depressive Disorder/ OR depressive disorder, major/ OR depressive disorder, treatment-resistant/ OR Mood Disorders/ OR (depression* OR depressive OR bipolar OR "mood disorder*" OR "affective disorder*").ab,ti,kf.))) NOT (Animals/ NOT Humans/)

APA PsycInfo Ovid

APA PsycInfo 1806 to September 2024 Week 3
784 results on 24 September 2024

(((*deep brain stimulation/ OR "deep brain stimul*".ab,ti.) AND (*treatment resistant depression/ OR depress*.ti. OR (resistant ADJ2 depress*).ab,ti.)) OR ((deep brain stimulation/ OR transcranial magnetic stimulation/ OR (("deep brain" ADJ3 stimul*) OR DBS OR "brain depth stimul*" OR

"intracranial brain stimul*" OR "transcranial magnetic stimul*" OR TMS OR rTMS OR "theta burst stimul*" OR TBS).mp.) AND (multitarget* OR ((multiple OR multi OR dual OR combined OR several) ADJ3 (target* OR stimulation)) OR (bilateral ADJ6 stimul*) OR (bilateral ADJ3 (DBS OR TBS OR rTMS OR EpCS OR implentation*)) OR "ventral capsule/ventral striatum").mp. AND (major depression/ OR endogenous depression/ OR reactive depression/ OR recurrent depression/ OR treatment resistant depression/ OR affective disorders/ OR (depression* OR depressive OR bipolar OR "mood disorder*" OR "affective disorder*").ab,ti.)))

Cochrane Database of Systematic Reviews Wiley

Issue 9 of 12, September 2024
1 results on 24 September 2024

((("deep brain" NEXT stimul*):ab,ti,kw AND (depress*:ti OR (resistant NEXT/2 depress*):ab,ti,kw)) OR ((("deep brain" NEXT/3 stimul*) OR DBS OR ("brain depth" NEXT stimul*) OR ("intracranial brain" NEXT stimul*) OR ("transcranial magnetic" NEXT stimul*) OR TMS OR rTMS OR ("theta burst" NEXT stimul*) OR TBS):ab,ti,kw AND (multitarget* OR ((multiple OR multi OR dual OR combined OR several) NEXT/3 (target* OR stimulation)) OR (bilateral NEXT/6 stimul*) OR (bilateral NEXT/3 (DBS OR TBS OR rTMS OR EpCS OR implentation*)) OR "ventral capsule/ventral striatum"):ab,ti,kw AND (depression* OR depressive OR bipolar OR (mood NEXT disorder*) OR (affective NEXT disorder*)):ab,ti,kw))

Cochrane Central Register of Controlled Trials Wiley

Issue 8 of 12, August 2024
511 results on 24 September 2024

((("deep brain" NEXT stimul*):ab,ti,kw AND (depress*:ti OR (resistant NEXT/2 depress*):ab,ti,kw)) OR ((("deep brain" NEXT/3 stimul*) OR DBS OR ("brain depth" NEXT stimul*) OR ("intracranial brain" NEXT stimul*) OR ("transcranial magnetic" NEXT stimul*) OR TMS OR rTMS OR ("theta burst" NEXT stimul*) OR TBS):ab,ti,kw AND (multitarget* OR ((multiple OR multi OR dual OR combined OR several) NEXT/3 (target* OR stimulation)) OR (bilateral NEXT/6 stimul*) OR (bilateral NEXT/3 (DBS OR TBS OR rTMS OR EpCS OR implentation*)) OR "ventral capsule/ventral striatum"):ab,ti,kw AND (depression* OR depressive OR bipolar OR (mood NEXT disorder*) OR (affective NEXT disorder*)):ab,ti,kw))

Web of Science Core Collection

Science Citation Index Expanded (1900-present), Social Sciences Citation Index (1900-present), Arts & Humanities Citation Index (1975-present), Conference Proceedings Citation Index-Science (1990-present), Book Citation Index (2005-present), Emerging Sources Citation Index (2005-present), Current Chemical Reactions and Index Chemicus

Advanced search > More options > Exact search

1878 results on 24 September 2024

((TS=("deep brain stimul*") AND (TI=(depress*) OR TS=(resistant NEAR/1 depress*))) OR TS=((("deep brain" NEAR/2 stimul*) OR DBS OR "brain depth stimul*" OR "intracranial brain stimul*" OR "transcranial magnetic stimul*" OR TMS OR rTMS OR "theta burst stimul*" OR TBS) AND (multitarget* OR ((multiple OR multi OR dual OR combined OR several) NEAR/2 (target* OR stimulation)) OR (bilateral NEAR/5 stimul*) OR (bilateral NEAR/2 (DBS OR TBS OR rTMS OR EpCS OR implentation*)) OR "ventral capsule/ventral striatum") AND (depression* OR depressive OR bipolar OR "mood disorder*" OR "affective disorder*"))) NOT DT=(Meeting Abstract)
